# Foot shape assessment techniques for orthotic and footwear applications: a methodological literature review

**DOI:** 10.3389/fbioe.2024.1416499

**Published:** 2024-09-05

**Authors:** Femke Danckaers, Kristina Stanković, Toon Huysmans, Brian G. Booth, Jan Sijbers

**Affiliations:** ^1^ imec - Vision Lab, Department of Physics, University of Antwerp, Antwerp, Belgium; ^2^ Section on Applied Ergonomics & Design, Department of Human-Centered Design, Delft University of Technology, Delft, Netherlands; ^3^ imec - Image Processing and Interpretation Lab, TELIN Department, Ghent University, Ghent, Belgium

**Keywords:** foot shape, foot assessment, techniques, measurements, foot modeling

## Abstract

**Introduction:**

Foot shape assessment is important to characterise the complex shape of a foot, which is in turn essential for accurate design of foot orthoses and footwear, as well as quantification of foot deformities (e.g., hallux valgus). Numerous approaches have been described over the past few decades to evaluate foot shape for orthotic and footwear purposes, as well as for investigating how one’s habits and personal characteristics influence the foot shape. This paper presents the developments reported in the literature for foot shape assessment.

**Method:**

In particular, we focus on four main dimensions common to any foot assessment: (a) the choice of measurements to collect, (b) how objective these measurement procedures are, (c) how the foot measurements are analyzed, and (d) other common characteristics that can impact foot shape analysis.

**Results:**

For each dimension, we summarize the most commonly used techniques and identify additional considerations that need to be made to achieve a reliable foot shape assessment.

**Discussion:**

We present how different choices along these two dimensions impact the resulting foot assessment, and discuss possible improvements in the field of foot shape assessment.

## 1 Introduction

The famous Greek philosopher Socrates once said “When our feet hurt, we hurt all over”, which underpins the importance of pursuing maximal foot comfort for overall satisfaction and healthier life. This comfort can be improved through adequate footwear and orthotic design, both of which depend on a foot shape assessment. A better understanding of how an individual’s characteristics, such as foot and leg alignment, influence foot shape may lead to improvements in the overall comfort and functionality of footwear and orthotic devices (Miller et al., 2000; Mundermann et al., 2001). Hence, a quantitative description of foot shape is crucial for a number of different applications related to the ergonomic design of footwear, foot orthotics and insoles, as well as clinical assessment of foot deformities.

Due to the foot’s complex anatomical structure, flexibility, and variety of geometric features, a number of measurement procedures have been developed for foot examination. Each procedure differs based on (a) the choice of measurements to collect, and (b) how these foot measurements analyzed in orthotic and footwear applications. Traditionally, a foot is examined visually by an experienced and well-trained expert (De Castro et al., 2011; de Castro et al., 2010a), often in different standing pose (half-weight bearing), sitting pose (non-weight bearing), or by placing the foot on plexiglass. However, visual assessment is highly objective and entirely relies on the knowledge and experience of the examiner. This has led experts to use different measurement equipment and devices such as sliding calipers, tapes, and 3D scanners (Bookstein and Domjanić, 2014; Hawes et al., 1992; Wang et al., 2018). Yet, this type of foot shape assessment is similarly inherently operator-dependent (Knippels et al., 2014). In order to eliminate human influence and objectify foot measurement, automated measurement procedures have been introduced using specialized devices (e.g., 3D scanners) that enable (semi-) automatic measurements of the foot shape (Cao et al., 2023; Zhang et al., 2023). The use of these technological inventions (e.g., laser 3D scanners, structured-light 3D scanners, plantar pressure measurement plates) have, in turn, given way to more advanced types of measurement data, which have spurred research into new data analysis techniques.

The variety of techniques for foot shape assessment is also exceedingly large (Allan et al., 2023). Common foot assessment methods compare foot shape between distinct populations, such as females and males (Saghazadeh et al., 2015; Fritz et al., 2013). These methods test for differences in key geometrical features (such as foot dimensions) between distinctive populations (Fritz et al., 2013). Other, more advanced, techniques, such as principal component analysis, analyse the entire foot shape based on its geometry, rather than individual measurements of different foot dimensions (Stanković et al., 2018; Stanković et al., 2020). In addition, personal characteristics or lifestyle choices can be linked to the foot shape, and their impact on foot shape can be measured. Knowledge about the relation between these factors and foot shape is often used to determine the predictive significance and generate prediction models (Stanković et al., 2020). These analyses are frequently achieved through regression analysis where foot shape is examined through the estimation of specific foot shape characteristics (Stanković et al., 2018; Fritz et al., 2013). Finally, techniques have been proposed to examine the complex shape of a foot by categorizing it into distinctive groups (Lee and Wang, 2015; Hong et al., 2011). This large variety of analysis techniques, coupled with advancements in measurement equipment, and measurement procedures for foot assessment, suggest that this review of the relevant literature is needed.

The purpose of this paper is to summarize the numerous ways foot shape has been assessed for orthotic and footwear design in the hopes of providing new researchers with an understanding of what considerations need to be made when designing a study of foot shape. In particular, we review various ways to measure foot shape, the levels of automation in these measurement procedures, the approaches to analyze the measurement data, and the various additional factors that can impact a foot shape analysis. Together, these four dimensions capture the main questions surrounding a foot shape assessment: what to measure (completeness), how to measure (accuracy and reliability), what can we get out of the measurements (types of analyses), and what else can impact our assessment (other factors). By reviewing the literature across these four dimensions, we hypothesize that we can provide a complete picture of how foot shape assessment is typically done, and identify the key considerations that need to be made to ensure a quality assessment of foot shape.

## 2 Methods

**FIGURE 1:**
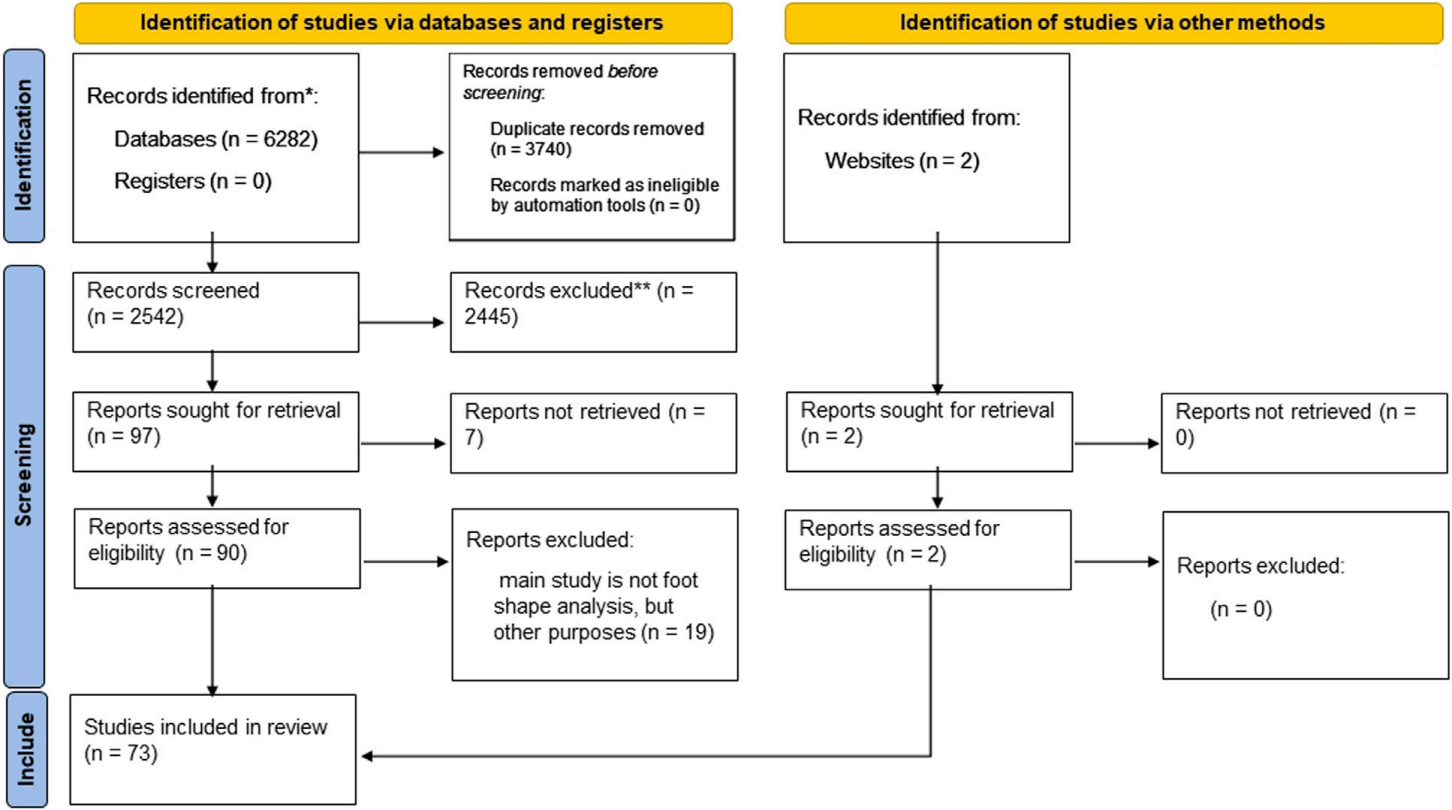
Flow of information: Different phases of our methodological literature review.

Our methodological literature review follows the best-practices laid out in (Aguinis et al., 2023), thereby following four main steps (summarized in Figure 1): study identification, study screening, study eligibility, and study inclusion.

### 2.1 Study identification

The initial literature search for this review was carried out in 2021/2022 in the PubMed and Scopus databases. The search was then independently repeated in April 2024 by a second researcher. In the identification phase, our inclusion criteria were chosen to identify studies that analyse the adult foot shape in orthopaedic and footwear applications. This meant that any paper in our review had to have (a) a mention of the foot or feet, (b) a mention of shape, (c) a mention of analysis or modelling, and (d) a mention of orthotics or footwear. These four criteria were then translated into the following keyword search string: *“(foot OR feet) AND (form OR forms OR shape OR shapes OR posture) AND (analysis OR analyses OR analysed OR model OR models OR modelling OR index OR score OR scale) AND (orthopaedic or orthopaedics OR orthotic OR orthotics OR orthosis OR orthoses OR footwear OR shoe) AND (papers after 1990) AND (English)”*. We remark that controlled vocabulary was not used in our search; only free text searches were used, which may have resulted in not all relevant articles being identified.

### 2.2 Study screening, eligibility, and inclusion

In the screening phase, all abstracts were reviewed. The first and second author read and assessed the full-text articles extracted from the screening phase. Articles were removed at the screening phase if they did not relate to foot shape (e.g., they mention the foot and shape in passing with the focus of the article being on something else) or did not assess the foot as a whole (e.g., an article that analyzes only the calcaneus). Additionally, to keep the review move focused, articles were removed if they did not focus on the adult age range. Articles were then considered for inclusion if their main focus was the analysis of foot shape for footwear and orthotic applications. Full inclusion and exclusion criteria are summarized in Figure 1.

### 2.3 Risks and biases

During our search for papers, we defined our search string so it covered a broad area. Afterwards, we excluded many papers because they were out of scope. This was done to reduce the risk of missing relevant studies. Furthermore, we checked for author bias, and the most frequently occurring authors are Menz (5/73 studies) and Nigg (5/73 studies). Therefore, we regard our study as not biased towards one specific author or research group.

## 3 Results

### 3.1 Search results

73 unique articles were screened that met the eligibility criteria (Figure 1). Two of them were identified from ScienceDirect, namely, (Knippels et al., 2014; Bogdan et al., 2017).

### 3.2 Choice of foot measurements

**FIGURE 2:**
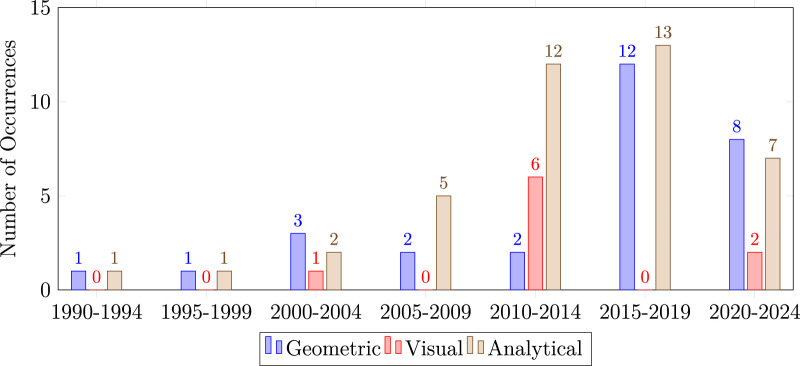
Histogram that shows the measurement trends over the years. Geometric measurement techniques, such as 3D scanning, gains popularity over the last years, while there is a decrease noticeable of objective observations.

Although several requirements must be met for a quality foot shape assessment, perhaps the most important is the choice of foot measurements to be collected. The studies we reviewed examined foot shape for different purposes (Domjanic et al., 2013; 2015; Stanković et al., 2018; Bookstein and Domjanić, 2014; Hawes et al., 1992; Conrad et al., 2019; Wang et al., 2018; Chen et al., 2018; Rijal et al., 2018; Chun et al., 2017; Price and Nester, 2016; Baek and Lee, 2016; Saghazadeh et al., 2015; Lee et al., 2015; Lee and Wang, 2015; Fritz et al., 2013; Krauss et al., 2011; Hong et al., 2011; Mickle et al., 2010; Krauss et al., 2008; Kouchi and Tsutsumi, 1996; Kim and Do, 2019; Echeita et al., 2016; Shu et al., 2015; Chiou et al., 2015; De Castro et al., 2011; de Castro et al., 2010a; de Castro et al., 2010b; Luo et al., 2009; Xiong et al., 2008; Stolwijk et al., 2013; Stanković et al., 2020; Booth et al., 2019; Swedler et al., 2010; Menz et al., 2012; Garrow et al., 2001; Thomson, 1994; Wunderlich and Cavanagh, 2001; Bob-Manuel and Didia, 2009; Jurca et al., 2019; Tsung et al., 2003; Young, 2020; Castro et al., 2010; Park and Kent, 2020; Jelen et al., 2005; Sforza et al., 1998; Barton et al., 2010; Levinger et al., 2010; Oladipo et al., 2009; Hill et al., 2017; Ballester et al., 2019; Alcacer et al., 2020; Cowley and Marsden, 2013; Wu et al., 2018; Maiwald et al., 2018; Ma and Luximon, 2014; Luximon and Goonetilleke, 2004; Mochimaru et al., 2000; Sun et al., 2009; Rogati et al., 2019; Huang et al., 2018; Cabero et al., 2021; Hu et al., 2018; Boppana and Anderson, 2021; Cao et al., 2023; Zhang et al., 2023; Schuster et al., 2021; Bogdan et al., 2017; Zhao et al., 2020; Yuan et al., 2021; Rogati et al., 2021; Allan et al., 2023) but, in general, foot shape was assessed by extracting important geometrical features using various measurement procedures. We observed three main approaches to the collection of foot measurements: qualitative (e.g., foot posture index, visual assessment), anthropometric (e.g., lengths, angles, circumferences, indexes), and geometric (e.g., marker locations, boundary curves, surfaces). These studies are shown according to date of publication in Figure 2.

A qualitative foot assessment is a purely visual appraisal, which relies entirely on the expertise of the examiner. Such studies make up 15% of those reviewed (11 of 73). These visual appraisals are often reported using numerical scales like the foot posture index (FPI) (Evans et al., 2003), visual assessment of the plantar surface to estimate foot arch height (Swedler et al., 2010), foot arch index (Menz et al., 2012), and hallux valgus scale (Garrow et al., 2001). Of these approaches, the FPI measure is the broadest, consists of summing the values of six different assessment criteria, where each criterion is scored visually on a scale from −2 to +2. This leads to a numerical foot shape score ranging from −12 (indicating maximal supination) to +12 (indicating maximal pronation) (De Castro et al., 2011; de Castro et al., 2010a; de Castro et al., 2010b; Young, 2020; Barton et al., 2010; Levinger et al., 2010; Cowley and Marsden, 2013). Other qualitative foot assessment mainly focus on one aspect of foot shape, be it arch height (Swedler et al., 2010; Menz et al., 2012) or hallux angle (Garrow et al., 2001). Although these visual assessment techniques do not quantitatively measure foot shape, their numerical scoring systems have the potential to be analyzed using quantitative algorithms.

**FIGURE 3:**
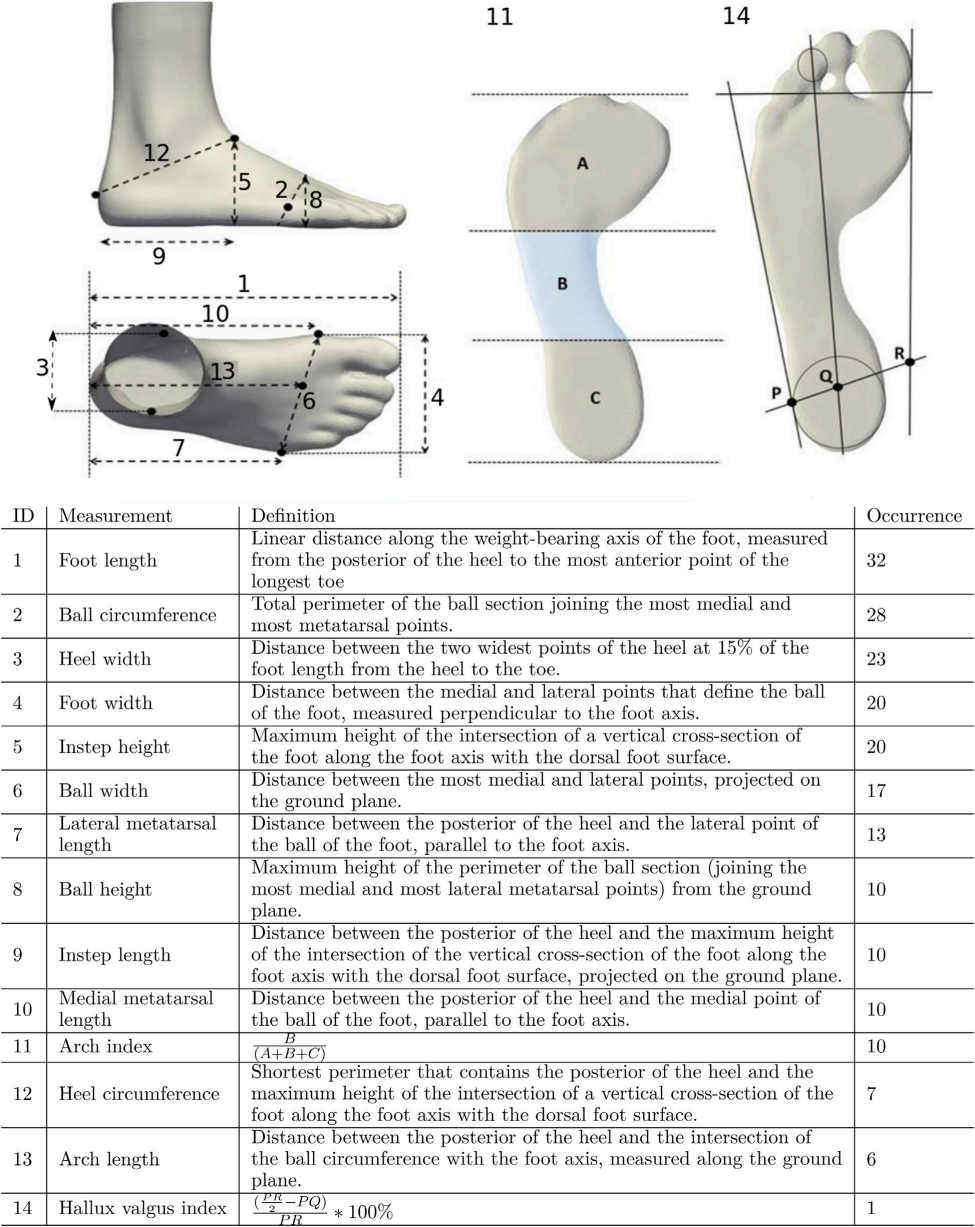
Quantitative metrics for assessing foot shape, ranked by occurrence in the literature (number of studies shown on the right). A, B and C denote, respectively, the areas of the forefoot, midfoot and heel of a footprint. P, Q and R denote lateral malleolus, center of the heel, and medial malleolus, respectively, on a line through the malleolus.

The second, and most popular, approach to foot measurement is represented in studies that use anthropometric measurements. Such studies make up 63% of those reviewed (46 of 73). These approaches collect one or more numerical measurements obtained as distinctive foot dimensions such as lengths, widths, angles, girths, heights, circumferences (Stanković et al., 2018; Bookstein and Domjanić, 2014; Hawes et al., 1992; Wang et al., 2018; Chen et al., 2018; Rijal et al., 2018; Chun et al., 2017; Price and Nester, 2016; Baek and Lee, 2016; Saghazadeh et al., 2015; Lee et al., 2015; Lee and Wang, 2015; Fritz et al., 2013; Krauss et al., 2011; Hong et al., 2011; Mickle et al., 2010; Krauss et al., 2008; Kouchi and Tsutsumi, 1996; Kim and Do, 2019; Echeita et al., 2016; Shu et al., 2015; De Castro et al., 2011; de Castro et al., 2010a; b; Wunderlich and Cavanagh, 2001; Barton et al., 2010; Levinger et al., 2010; Oladipo et al., 2009; Ballester et al., 2019; Wu et al., 2018; Maiwald et al., 2018; Huang et al., 2018; Cao et al., 2023; Zhang et al., 2023; Limon et al., 2023; Chertenko and Booth, 2022) or specific foot parameters such as arch index (Hawes et al., 1992; Chun et al., 2017; De Castro et al., 2011; de Castro et al., 2010a; b; Stolwijk et al., 2013; Levinger et al., 2010), or valgus index (Thomson, 1994). Figure 3 shows the anthropometric measurements most commonly seen in the reviewed studies. For an exhaustive list, we refer the reader to Table 1 of the Supplementary Material.

**TABLE 1:** Studies that are using different automation levels of measurement procedure related to the number of measured foot shape characteristics.

	Manual	Semi-automatic	Automatic
Foot lengths	10	22	6
Foot widths	13	19	6
Foot heights	16	18	6
Foot circumferences	10	14	5
Foot angles	7	13	2
Foot indexes	10	2	3
Plantar surface			11
3D foot shape			16

These anthropometric foot dimensions are most commonly measured using widely-available measurement equipment such as measuring tapes and sliding calipers. However, there are some exceptions. For example, arch index, which is the fraction of a footprint occupied by the midfoot, is obtained from footprints are generated manually using clay moulds (Hawes et al., 1992; De Castro et al., 2011; de Castro et al., 2010a; de Castro et al., 2010b; Sforza et al., 1998; Levinger et al., 2010), or automatically using foot-arch analysis platforms (Chun et al., 2017; Stolwijk et al., 2013; Chertenko and Booth, 2022). Similarly, the valgus index is defined as a measurement of the medial malleolar shift of the intermalleolar diameter in relation to the posterior foot support area (Thomson, 1994). It is calculated numerically from the footprints as shown in Figure 3. By collecting these foot dimensions and/or foot parameters, a complex foot shape can be well captured by a small number of numerical values in an objective way, convenient for further analysis.

The remaining 22% of reviewed studies (16 of 73) use a geometrical representation of the foot, where some parts of the foot shape (such as footprint outline), or the whole 3D foot shape, are analyzed holistically. These geometrical forms are digitally represented as a set of 2D or 3D points generated using clay moulds or 3D scanners, respectively. Usually, geometrically-significant discrete points are manually marked as a set of disconnected markers located on specific anatomical locations of the foot (Domjanic et al., 2013; 2015; Stanković et al., 2018; Bookstein and Domjanić, 2014; Conrad et al., 2019; Tsung et al., 2003; Park and Kent, 2020; Alcacer et al., 2020; Mochimaru et al., 2000; Cao et al., 2023). In (Domjanic et al., 2013; Domjanic et al., 2015; Bookstein and Domjanić, 2014), the footprint and foot outline were represented as curves using 85 markers and automatically derived pseudo-markers, while in the study of (Park and Kent, 2020), the entire 3D foot surface was represented by 240 pseudo-markers. Compared to those studies (Domjanic et al., 2013; Domjanic et al., 2015; Bookstein and Domjanić, 2014; Park and Kent, 2020; Alcacer et al., 2020), which all employ a limited number of markers, additional works in (Stanković et al., 2018; Conrad et al., 2019; Stanković et al., 2020; Booth et al., 2019; Jurca et al., 2019; Ma and Luximon, 2014; Luximon and Goonetilleke, 2004; Cao et al., 2023; Zhang et al., 2023; Chertenko and Booth, 2022) represented the foot surface at higher resolutions using all points obtained from the 3D scanner.

### 3.3 Measurement objectivity and automation

**FIGURE 4:**
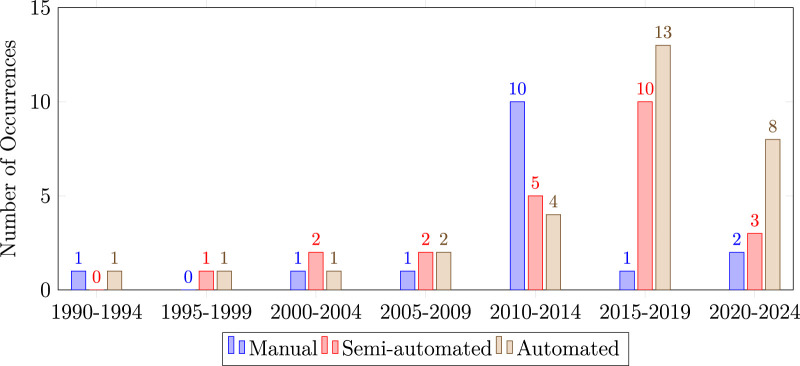
Histogram that shows the measurement trends over the years. Automated and semi-automated measurement techniques, such as 3D scanning, gains popularity over the last years, while there is a decrease noticeable of manual measurement techniques.

One aspect to consider in foot shape assessment is whether these shape measurements are collected in an objective and repeatable way. These aspects of objectivity and repeatability have been linked to how automated the measurement procedure is (Rogati et al., 2021). Therefore, in this section, we examine to what level the various foot shape measurement procedures are automated. Specifically, each reviewed measurement procedure was classified as either manual, semi-automatic, or automatic (Table 1) These studies are also shown according to date of publication in Figure 4.

#### 3.3.1 Manual

Manual foot shape quantification has been reported in 23% of reviewed studies (17 of 73) (Hawes et al., 1992; Wang et al., 2018; Chun et al., 2017; Echeita et al., 2016; De Castro et al., 2011; de Castro et al., 2010a; b; Wunderlich and Cavanagh, 2001; Bob-Manuel and Didia, 2009; Young, 2020; Castro et al., 2010; Barton et al., 2010; Levinger et al., 2010; Cowley and Marsden, 2013; Limon et al., 2023; Rogati et al., 2021). Manual assessment requires at least one experienced and well-trained professional to perform the measurements. In some cases, the experts visually examine the foot shape, for example, by visual categorization of foot shape based on arch height (De Castro et al., 2011; de Castro et al., 2010a; de Castro et al., 2010b; Swedler et al., 2010; Menz et al., 2012; Garrow et al., 2001; Young, 2020; Castro et al., 2010; Levinger et al., 2010). However, the more common approach to foot shape measurement is through numerical representation of foot shape characteristics (e.g., foot dimensions). Usually, the numerical foot measurements are obtained using devices such as: a ruler for measuring the foot arch height (Chun et al., 2017; Barton et al., 2010; Levinger et al., 2010); a Brannock device for measuring the foot length (Echeita et al., 2016; Boppana and Anderson, 2021; Limon et al., 2023); sliding caliper for measuring ball width (Echeita et al., 2016; Limon et al., 2023), foot length/width (De Castro et al., 2011; de Castro et al., 2010a; de Castro et al., 2010b; Bob-Manuel and Didia, 2009; Oladipo et al., 2009), and arch height (Hawes et al., 1992); a measuring tape for various foot circumferences (ball, low-instep, high-instep, heel instep, etc.) (Echeita et al., 2016; De Castro et al., 2011; de Castro et al., 2010a; de Castro et al., 2010b; Castro et al., 2010); a goniometer for angle measurements (De Castro et al., 2011; de Castro et al., 2010a; b; Castro et al., 2010; Barton et al., 2010); an analog pachymeter for foot widths and lengths (Castro et al., 2010); or an analog height rod for foot heights (Castro et al., 2010).

#### 3.3.2 Semi-automatic

Of the reviewed studies, 33% (24 of 73) use semi-automatic foot shape measurement techniques (Table 2). These methods extract numerical measurements from a digital representation of the foot such as a 2D image or a 3D optical scan. Prior to an automated measurement step, important features of the foot are manually annotated by placing markers at significant anatomical locations. These markers are placed either directly on the foot (physical markers) (Chen et al., 2018; Saghazadeh et al., 2015; Lee et al., 2015; Lee and Wang, 2015; Hong et al., 2011; Mickle et al., 2010; Krauss et al., 2008; Kouchi and Tsutsumi, 1996; Kim and Do, 2019; Shu et al., 2015; Luo et al., 2009; Xiong et al., 2008; Tsung et al., 2003; Hill et al., 2017; Alcacer et al., 2020; Maiwald et al., 2018; Mochimaru et al., 2000; Cao et al., 2023) or after the digital version of foot shape is obtained (virtual markers) (Rijal et al., 2018; Fritz et al., 2013; Krauss et al., 2011; Hu et al., 2018; Zhang et al., 2023; Bogdan et al., 2017). In the latter case, virtual markers are placed at significant anatomical locations on the digital foot representation using a variety of software tools [e.g., ScanWorX in the study of (Krauss et al., 2011), Geomagic in the study of (Fritz et al., 2013; Zhang et al., 2023), or D+ in the study of (Rijal et al., 2018)]. The markers on the foot, either physical or virtual, are then used to calculate important foot measurements (e.g., foot length, foot width) and important foot parameters (e.g., arch index). These subsequent calculations are thus obtained automatically.

**TABLE 2:** Different types of foot measurements and the most common research topics for different orthotic and footwear applications: Foot variability (i.e., study of the main shape variations of the foot), subject features (i.e., study of the relationship between different foot measurements), subject behaviour or health condition (i.e., study of influence of behaviour or health on the shape of the foot), custom products (i.e., study to improve footwear products), definition of product sizes (i.e., study to improve the footwear sizes), new measurement system (i.e., study to improve the measuring system of feet.).

	Foot variability	Subject features	Subject behaviour or health condition	Custom products	Definition of product sizes	New measure. System	Other
Foot length	6	10	5	4	2	3	2
Ball circumference	3	12	5	3	1	2	2
Heel width	1	12	4	2	1	1	2
Instep height	3	10	3	2	1	1	
Ball width	2	9	3		1	1	1
Lateral metatarsal length		8	1	1	2	1	1
Ball height		7	3				
Instep length		6	1	1	1		1
Medial metatarsal length		6		1	1	1	1
Arch index	1	5	3			1	
Heel circumference		4	1	1			1
Arch length	2	1			1	2	
Hallux valgus index							1

#### 3.3.3 Fully automated

In fully automated procedures, the foot is assessed without human interaction and key anatomical points are automatically identified as foot shape markers (pseudo-markers). Such studies make up 44% of those reviewed (32 of 73). In (Price and Nester, 2016; Saghazadeh et al., 2015; Chiou et al., 2015; Park and Kent, 2020), only a certain number of pseudo-markers on the digital foot (such as markings of navicular tuberosity) were considered necessary to automatically obtain foot dimensions. In (Domjanic et al., 2013; 2015; Stanković et al., 2018; Conrad et al., 2019; Stanković et al., 2020; Jurca et al., 2019; Zhang et al., 2023), where the foot shape variations are assessed for given populations, the entire point cloud in 3D space was selected as the significant pseudo-markers.

Both semi-automatic and automatic procedures require 2D imaging systems (Stanković et al., 2018; Hong et al., 2011; Kouchi and Tsutsumi, 1996; Kim and Do, 2019; Shu et al., 2015; Stolwijk et al., 2013; Jelen et al., 2005; Sforza et al., 1998), 3D scanners (Domjanic et al., 2013; 2015; Conrad et al., 2019; Chen et al., 2018; Rijal et al., 2018; Price and Nester, 2016; Baek and Lee, 2016; Saghazadeh et al., 2015; Lee et al., 2015; Lee and Wang, 2015; Fritz et al., 2013; Krauss et al., 2011; Mickle et al., 2010; Krauss et al., 2008; Chiou et al., 2015; Xiong et al., 2008; Stanković et al., 2020; Booth et al., 2019; Jurca et al., 2019; Park and Kent, 2020; Alcacer et al., 2020; Wu et al., 2018; Maiwald et al., 2018; Ma and Luximon, 2014; Luximon and Goonetilleke, 2004; Sun et al., 2009; Rogati et al., 2019; Huang et al., 2018; Cabero et al., 2021; Hu et al., 2018; Mochimaru et al., 2000; Schuster et al., 2021; Zhao et al., 2020; Rogati et al., 2021), or dynamic 4D scanners (Fritz et al., 2013; Boppana and Anderson, 2021; Zhang et al., 2023). The 2D imaging systems can be further split into single-camera and multi-camera approaches. These systems are broadly used to acquire the plantar surface (Stanković et al., 2018; Kim and Do, 2019; Shu et al., 2015; Stolwijk et al., 2013; Jelen et al., 2005; Sforza et al., 1998; Chertenko and Booth, 2022), or to generate a static geometric foot representation (Hong et al., 2011; Kouchi and Tsutsumi, 1996; Schuster et al., 2021).

### 3.4 Techniques for foot shape analysis

Different approaches have been developed to analyse foot shape. Based on the purpose of the study, the following types of foot shape analysis can be distinguished: describing and modelling foot shape variations (shape variation studies), comparing foot shapes across groups (group studies), prediction of foot shape based on a limited set of easy to obtain measurements (prediction studies), and grouping individuals based on foot shape (clustering and classification). In this section, we review these common foot shape analysis techniques and where they are most commonly used.

Before examining each analysis technique in detail, we observed that the large variety of techniques for foot shape assessment has also led to a wide range of applications within the field of orthotics and footwear. The relationship between analysis techniques and subsequent applications are summarized Table 3. The results in this table suggest a clear interest towards working with groups, both in terms of comparing existing groups and in terms of defining subgroups through clustering and classification. Additionally, we compared analysis techniques to the choice of foot measurements in Table 1. A clear relationship was not visible there.

**TABLE 3:** Foot shape analysis methods for orthotic and footwear applications.

	Foot shape variations	Group comparisons	Prediction	Classification and clustering
Describe foot variation	7	1	2	4
Linking to subject characteristics	3	20	4	5
Linking to subject behaviour and health condition		12		
Custom products	1	2	1	2
Definition of product sizes	1	1	1	4
New measurement system	1	4		2

#### 3.4.1 Groups studies of foot shape

The reviewed literature revealed that foot shape is most commonly used to compare different populations. Of the 73 studies reviewed, 40 of them performed some sort of group comparison. This type of analysis was commonly seen when proposing new foot shape measurement procedures, and when examining foot shape differences between females and males.

When evaluating a new foot shape measurement procedure, measurements from the new technique are usually compared, over the same set of feet, to a similar measurement obtained from an established measurement procedure (Bookstein and Domjanić, 2014; Wang et al., 2018; Rijal et al., 2018; Chun et al., 2017; Zhang et al., 2023; Rogati et al., 2021). Usually, the measurement procedures are compared numerically by computing differences in foot shape using an established dissimilarity metric. Common dissimilarity metrics used here include: (root) mean squared error (MSE) between data (Wang et al., 2018; Wu et al., 2018; Ma and Luximon, 2014; Rogati et al., 2019; Boppana and Anderson, 2021); Spearman rank order correlation (Chun et al., 2017); data distribution parameters (Rijal et al., 2018); mean Euclidean distance between shapes (Wu et al., 2018); Hausdorff distance between shapes (Boppana and Anderson, 2021); linear regression (Lee et al., 2015); and mean shortest distance from predicted to actual shape (Luximon and Goonetilleke, 2004). Beside comparing the measurements directly, the distributions of the measurements are often compared as well in terms of their mean and standard deviation (with the normality of the distributions being assumed).

Group studies have also been used to compare foot shape characteristics between distinct populations such as females and males, or older and younger people. In these studies, differences between populations are exclusively examined through the use of statistical testing. Common testing strategies include: t-tests on foot dimensions (Chen et al., 2018; Saghazadeh et al., 2015; Lee et al., 2015; Lee and Wang, 2015; Fritz et al., 2013; Krauss et al., 2011; Hong et al., 2011; Mickle et al., 2010; Krauss et al., 2008; Chiou et al., 2015; De Castro et al., 2011; de Castro et al., 2010a; de Castro et al., 2010b; Luo et al., 2009; Xiong et al., 2008; Stolwijk et al., 2013; Wunderlich and Cavanagh, 2001; Bob-Manuel and Didia, 2009; Jurca et al., 2019; Tsung et al., 2003; Young, 2020; Sforza et al., 1998; Barton et al., 2010; Oladipo et al., 2009; Cao et al., 2023); Hotelling’s T-squared test (Maiwald et al., 2018); Pearson’s chi-squared test (Levinger et al., 2010); analysis of variance (ANOVA) (Price and Nester, 2016; Fritz et al., 2013; Shu et al., 2015; Ballester et al., 2019; Cowley and Marsden, 2013); or analysis of probability (de Castro et al., 2010a; de Castro et al., 2010b; Castro et al., 2010). The most common technique, the *t*-test, assumes that the foot shape measurements are (a) collected from a representative, randomly selected portion of the total population, and (b) the measurements are continuous and normally distributed.

#### 3.4.2 Modelling foot shape variations

Seven of the 73 reviewed studies (9.6%) (Domjanic et al., 2013; Domjanic et al., 2015; Stanković et al., 2018; Conrad et al., 2019; Park and Kent, 2020; Sforza et al., 1998) describe the anatomical variation in foot shape measurements for a given population. A convenient way to analyse these measurements is using Principal Component Analysis (PCA). In order to apply PCA, it is necessary to have shape data that have identical representations. In other words, the geometrical markers or measurements need to be brought into correspondence and, potentially, superimposed. A common way to do so is by using Generalized Procrustes Analysis (Domjanic et al., 2013; Domjanic et al., 2015; Stanković et al., 2018; Conrad et al., 2019). When PCA is applied to geometrical measurements, an average shape for a given population, and the main modes of variation from the mean shape, are obtained. Geometrically speaking, the main modes of variation, also referred to as principal components (PCs), represent the most common changes in foot shape and can be interpreted as shape deformations. Each foot shape can then be represented as the average foot shape plus the sum of weighted PCs. In this way, it is possible to visualize how much - and in which regions - foot shape varies the most.

Another convenient approach to describe variability of a footprint outline is Fourier analysis (Sforza et al., 1998). This method allows a quantitative analysis of a shape and of its changes independent from foot size. The Fourier coefficients can be standardized for size, position, and orientation and do not need external reference planes. The resulting coefficients are often referred to as Fourier descriptors.

Additionally, 6 of the 73 reviewed studies (8.2%) suggested examining how personal characteristics (e.g., age, body-mass index) influence foot shape variation (Domjanic et al., 2013; Domjanic et al., 2015; Stanković et al., 2018; Fritz et al., 2013; Echeita et al., 2016). The most common technique used for prediction of a dependent variable is multiple linear regression (MLR). MLR is based on the assumption that there is a linear relationship between both the dependent and independent variables. An alternative to MLR is principal component regression (PCR) (Stanković et al., 2020). PCR combines elements of PCA and MLR into a single analysis framework. It therefore relies on the same assumptions as in regular multiple regression: linearity, constant variance (no outliers), and independence.

#### 3.4.3 Prediction

A useful asset during the foot assessment would be to estimate the full foot shape based on a small number of personal characteristics or foot shape measurements. Of the 73 studies we reviewed, 8 studies (11%) perform this type of prediction. The most commonly-used technique to determine the predictive significance of personal characteristics on foot shape is multiple linear regression. Several studies employed this technique to develop prediction models (Hawes et al., 1992; Echeita et al., 2016; Chiou et al., 2015; Hill et al., 2017). Other prediction methods employ machine learning algorithms (Booth et al., 2019), which may yield notable improvement in terms of accuracy and efficiency.

Considering the shape dimensions of feet that are not always proportional to each other, another technique that has been employed is allometry (Xiong et al., 2008). Allometry is the study of the relative size of different parts of a body as a consequence of growth. In the context of foot shape analysis, this allometric model was used to investigate how foot shape dimensions change as foot size changes.

#### 3.4.4 Classification and clustering

Classification techniques are used to examine foot shape by assigning feet into predefined classes. Once a foot is assigned to a class, the knowledge of the shape characteristics of the whole class could be used in further analysis. Ten studies out of 73 (14%) employ this type of analysis. Of these classification approaches, the simplest relies on pure visual appraisal of the examiner (Swedler et al., 2010; Menz et al., 2012; Garrow et al., 2001).

Automated classification techniques have also been used in literature when assessing the foot shape (Chen et al., 2018; Mickle et al., 2010; Alcacer et al., 2020; Mochimaru et al., 2000; Limon et al., 2023). The simplest of these classification technique uses the mean and standard deviation of the foot measurements as the limits between the classes (Hu et al., 2018; Limon et al., 2023). Similarly, the location of central tendency has also been used (Sun et al., 2009). Furthermore, free form deformations have been used in classification to capture the dissimilarity between two 3D foot shapes (Mochimaru et al., 2000).

A promising classification technique for establishing typologies of foot shape is an archetypoid analysis (ADA) (Alcacer et al., 2020). In general, ADA is an extended variant of archetype analysis applied to shapes with landmarks. The objective of ADA is to represent the cases by means of a mixture of representative archetypoids. Thus, the results returned by ADA easily interpretable, even for non-experts. Archetype analysis has also been combined with the k-Nearest Neighbor algorithm to detect extreme shape anomalies (Cabero et al., 2021).

Another common technique used for classification is discriminant analysis (Chen et al., 2018; Wunderlich and Cavanagh, 2001). In many ways, discriminant analysis parallels multiple regression analysis. The main difference between these two techniques is that regression analysis deals with continuous dependent variables, while discriminant analysis must have discrete dependent variables.

Similar to classification, clustering identifies similarities between objects, and groups them according to common characteristics, while also differentiating them from other groups of objects. The main difference between classification and clustering is that the former uses pre-defined groups while the latter discovers the groups present in the data. Clustering algorithms have been employed in 6 of the 73 studies we reviewed (8.2%).

The most common clustering technique is K-means clustering. The goal of this technique is to group data points into distinct non-overlapping subgroups. In general, the K-means algorithm identifies k centroids and then, in an iterative procedure, allocates every data point to the nearest cluster while also optimizing the positions of the centroids. This approach is used in two-stage cluster analysis for foot type classification in multiple studies (Lee and Wang, 2015; Hong et al., 2011; Krauss et al., 2008; Kim and Do, 2019; Huang et al., 2018). Prior to K-means analysis the number of clusters (k) for subsequent cluster analysis needs to be determined. This is obtained using Ward’s minimum variance method (Ward Jr, 1963), in which the total within-cluster variance is minimized using a recursive algorithm.

Another approach for clustering is presented in the study of Baek and Lee (Baek and Lee, 2016). Here, hierarchical clustering is used to categorize feet using geometrical foot shape measurements. The differences in foot shape between obtained clusters were validated visually as well as numerically by calculating mean, std, min, max of foot dimensions obtained from each group’s mean shapes. This clustering approach is much more advanced than conventional foot-shape classification methods in that it takes into account the comprehensive geometry of the entire foot.

### 3.5 Influence of subject characteristics and subject behaviour

As hinted at earlier, different lifestyle choices (e.g., frequency of sport activity, shoe wearing habits) and personal characteristics (e.g., sex, body mass index, age, ethnicity) have been shown to significantly influence foot morphology (Domjanic et al., 2013; Domjanic et al., 2015; Stanković et al., 2018; Bookstein and Domjanić, 2014; Chen et al., 2018; Price and Nester, 2016; Saghazadeh et al., 2015; Lee et al., 2015; Lee and Wang, 2015; Fritz et al., 2013; Krauss et al., 2011; Hong et al., 2011; Mickle et al., 2010; Krauss et al., 2008; Echeita et al., 2016; Chiou et al., 2015; De Castro et al., 2011; Luo et al., 2009; Xiong et al., 2008; Stolwijk et al., 2013; Wunderlich and Cavanagh, 2001; Bob-Manuel and Didia, 2009; Jurca et al., 2019; Tsung et al., 2003; Barton et al., 2010; Oladipo et al., 2009; Cao et al., 2023). In fact, multiple group studies in Section 3.4.1 show differences related to personal habits or characteristics. In total, 27 of the 73 reviewed studies (37%) have evaluated the influence of lifestyle choices and personal characteristics on foot shape. These studies are summarized in Table 4, where each personal factor is linked to the foot region whose morphology is impacted. As a result of these studies, the impact of these factors should also be taken into consideration when performing an analysis of foot shape.

**TABLE 4:** Number of papers showing which factors influence specific foot regions.

	Age	BMI	Ethnicity	Foot problems	Sex	Others
Toes	2		1	Hallux valgus, Toe deformity, Swollen foot	6	High-heeled shoes, Frequency of sport activity, Different bearing weight, Shod and unshod runners
Forefoot	1	5	3	Hallux valgus, Toe deformity, Swollen foot	9	High-heeled shoes, Geographic region, Frequency of sport activity, Different bearing weight, Shod and unshod runners
Midfoot	2	8	2	Hallux valgus, Toe deformity, Patellofemoral pain syndrome, Osteoarthritis	13	Frequency of sport activity, Geographic region
Heel	1	3	1	Swollen foot, Patellofemoral pain syndrome, Arthritis, Osteoarthritis, Diabetic foot	5	
Ankle	1	2	1	Toe deformity, Swollen foot, Patellofemoral pain syndrome, Arthritis, Osteoarthritis, Diabetic foot	4	Frequency of sport activity

Age: Six studies (8.2%) examined the influence of age on foot shape. No significant relationship was found when the effect of the age was evaluated on the footprint shape (Domjanic et al., 2013) and on 4D dynamic foot scans (Fritz et al., 2013). In contrast to these findings, other studies reported changes in older populations: a wider heel, a less noticeable Achilles tendon, a more visible hallux valgus, and thicker toes (Stanković et al., 2018); flatter, wider feet (Echeita et al., 2016); and greater volume in the forefoot for older female populations (De Castro et al., 2011). These insights should be considered when designing shoes for older populations (Bogdan et al., 2017).

Body-Mass Index (BMI): Seven studies (9.6%) examined the influence of BMI on foot shape. Increased BMI is associated to many foot shape changes: wide and flat feet (Domjanic et al., 2013; Domjanic et al., 2015); thicker forefoot along the dorsoplantar axis, a wider Achilles tendon, a wider heel, and a wider ankle (Stanković et al., 2018); wider foot, wider ball, bigger ball circumference, lower ball height, wider heel, and bigger heel circumference (Price and Nester, 2016); wider midfoot, and a more pronounced changes of medial ball length and ball width during stance phase (Fritz et al., 2013); bigger instep circumference (Echeita et al., 2016); longer foot, wider foot, and a decrease of arch height (Chiou et al., 2015). Based on the findings reported in the above studies, it can be concluded that foot shape changes significantly as BMI changes.

Ethnicity: The influence of ethnicity on foot shape was evaluated in 5 of the reviewed studies (6.8%). A significant difference in forefoot shape between Taiwanese and Japanese females was reported in (Lee et al., 2015). Wider feet were also noted in Taiwanese adults compared to Mainland Chinese and Europeans (Lee and Wang, 2015). Stolwijk et al. (2013) reported a lower arch index for most Malawian subjects, compared to Dutch subjects. The foot dimensions of Nigerian population were also found to be comparatively larger than Caucasian ones (Bob-Manuel and Didia, 2009). These findings match with the theoretical expectation that populations living in warm climates would have longer arms and legs than populations living in cold environments (Scherider, 1975). Large foot dimensions are an adaptation to tropical environments as they increase the surface area available for heat loss (Scherider, 1975).

Foot problems: Seven studies (9.6%) examined how the foot shape of people without foot problems differs from the foot shape of those with problems (e.g., hallux valgus). As expected, foot problems can have a significant impact on foot shape. Stankovic et al. (2020) reported that the biggest toe and head of the first metatarsal bone were the main regions of deviation for hallux valgus subjects, compared to the healthy foot shape. The study by Mickle et al. (2010) indicated that subjects who have moderate to severe hallux valgus feet had a significantly increased ball girth, ball width, medial and lateral ball lengths, heel bone angle, and first toe angle. They also reported that the individuals with swollen feet had a significantly increased ball girth, ball width and heel width, likely due to the excess fluid present in the foot region. It was stated that individuals with lesser toe deformities displayed an increased first and fifth toe height, first toe angle, and medial ball length. In addition, these subjects also showed a decreased ball height, medial malleoli height, navicular height and instep height compared to those without lesser toe deformities (Mickle et al., 2010). The study by Barton et al. (2010) reported that those with Patellofemoral Pain Syndrome (PFPS) showed significant differences in FPI, normalized navicular drop, and calcaneal angle (relative to the subtalar joint) compared to control groups. Significant differences in the FPI were also found in the study by Castro et al. (2010) where they examined the differences in foot shape of women with and without arthritis. The study by Levinger et al. (2010) indicated significant differences between control and medial compartment knee osteoarthritis (OA) groups in relation to the FPI, navicular drop, and the arch index. Finally, the study by Young (Young, 2020) reported significant differences in FPI between people having Charcot foot and subjects without any diabetic complication.

Sex: Seventeen studies (23%) discuss sex related differences in foot morphology (Domjanic et al., 2015; Stanković et al., 2018; Saghazadeh et al., 2015; Lee et al., 2015; Lee and Wang, 2015; Fritz et al., 2013; Krauss et al., 2011; Hong et al., 2011; Mickle et al., 2010; Krauss et al., 2008; De Castro et al., 2011; Cao et al., 2023). It has been reported that the female foot shape is characterized as having: relatively narrow footprint (lower width-to-height ratio), smaller distal toe elements, and a higher arch (Domjanic et al., 2015); a narrower ankle width, a hallux valgus, a narrower Achilles tendon, and a narrower heel compared to the male foot (Stanković et al., 2018); greater first toe angle (Saghazadeh et al., 2015); greater first and fifth metatarsophalangeal angles (De Castro et al., 2011); smaller ball girth (Saghazadeh et al., 2015; Lee and Wang, 2015; Xiong et al., 2008; Cao et al., 2023); smaller instep girth (Saghazadeh et al., 2015; Lee and Wang, 2015; Wunderlich and Cavanagh, 2001; Cao et al., 2023); lower instep height (Saghazadeh et al., 2015; Lee and Wang, 2015; Jurca et al., 2019); lower navicular height (Saghazadeh et al., 2015; Wunderlich and Cavanagh, 2001; Cao et al., 2023); narrower foot breadth (Lee and Wang, 2015; Luo et al., 2009; Bob-Manuel and Didia, 2009; Jurca et al., 2019; Xiong et al., 2008; Cao et al., 2023); narrower heel breadth (Lee and Wang, 2015; Jurca et al., 2019; Cao et al., 2023); larger ankle girth (Wunderlich and Cavanagh, 2001); shallower first toe (Wunderlich and Cavanagh, 2001); shorter ankle length (Wunderlich and Cavanagh, 2001); ball length (Luo et al., 2009; Zhang et al., 2023); greater ball circumference (Lee and Wang, 2015); narrower widths (Krauss et al., 2011; Hong et al., 2011; Mickle et al., 2010; Krauss et al., 2008; Cao et al., 2023); smaller girths (Hong et al., 2011; Mickle et al., 2010; Cao et al., 2023); and lower heights (Krauss et al., 2011; Hong et al., 2011; Mickle et al., 2010; Krauss et al., 2008; Cao et al., 2023) compared to men’s feet. The study of Saghazadeh et al. (2015) reported a greater foot arch height for males, which is contrary to other findings (Domjanic et al., 2015; Stanković et al., 2018; Wunderlich and Cavanagh, 2001). There were no significant differences reported between gender when examining dynamic foot shape (Fritz et al., 2013), or arch index and foot posture index (De Castro et al., 2011).

Lifestyle: Finally, factors that reflect lifestyle habits on the foot shape have been evaluated in 5 of the reviewed studies (6.8%). Wearing high-heeled shoes is associated with a larger forefoot area in the footprint and a relatively long hallux (Domjanic et al., 2013). Meanwhile, the frequency of sport activity is not influencing the footprint shape (Domjanic et al., 2013), though it has been reported that more physically active people tend to have a more narrow Achilles tendon, a more narrow midfoot, and thicker toes (Stanković et al., 2018); a narrower foot (Chen et al., 2018); and longer toes (Chen et al., 2018) then less physically active people. The study of Shu (Shu et al., 2015) further reported significant differences in foot shape of shod and unshod runners for foot length, width, hallux angle, and the minimal distance from hallux to second toe. Finally, Cowley and Marsden (Cowley and Marsden, 2013) showed that arch height tended to decrease after running a half marathon.

## 4 Discussion

The goal behind this review was to provide new researchers with an understanding of the foot shape considerations that should be made when designing orthotics and footwear. With this in mind, we reviewed the literature across four dimensions fundamental to any foot shape assessment: what to measure; how to measure; how to analyze the measurements; and what additional factors can impact this analysis. Our results show that the field shows no consensus across any of these dimensions, nor any of the interactions between these dimensions. Therefore, a discussion is required on the nuances involved in the decisions made along these four foot shape analysis dimensions.

### 4.1 What to measure

The most popular approach to the measuring of foot shape is to collect anthropometric measurements like those listed in Figure 3. These types of measurements have their advantages in that they are usually easy to collect with low cost equipment (e.g., measuring tapes, sliding calipers). anthropometric measurements are also usually easy to interpret and are small enough in number to keep the quantitative analyses simple.

Nevertheless, there are noticeable concerns with the use of anthropometric measurements for foot shape analysis. Perhaps the most surprising concern with respect to these measurements is that a standardized set of anthropometric measurements is not used across studies. We observed different studies defining their own anthropometric foot shape measurements in slightly different ways [see Table 1 of the Supplementary Material). This habit persists even though there are ISO norms (e.g., ISO 19408 (Committee, 2015)] of the relevant anthropometric foot measurements to use for footwear design. This lack of consistency in the use of anthropometric foot measurements makes it challenging to compare different studies, or to extrapolate the conclusions of these studies of feet that have been measured in a slightly different way.

In addition to the lack of consistency in the measures themselves, the use of anthropometric foot measurements can be limited by the small number of measurements that are taken. While anthropometric foot measurements are often intuitive, well chosen, and well justified, they inherently summarize the complex and detailed foot shape in a way that inevitably discards some foot shape information. This effect is particularly noticeable when comparing the results from studies that use anthropometric foot measurements to studies that use 3D scans of the foot surface (Rogati et al., 2019). Studies that analyze the full 3D foot shape occasionally lead to different conclusions than those that use anthropometric foot measurements, including, for example, whether age impacts foot shape (Domjanic et al., 2013; Stanković et al., 2018). It is interesting to note that the number of studies that use higher-dimensional geometric measurements, like those taken with 3D scanners, is trending upward (Figure 2). This trend may indicate an understanding in the field that a small number of anthropometric foot measurements may not fully capture the relevant features of foot shape to design high-quality orthotics and footwear.

Finally, it is worth noting that qualitative foot assessments, while fewer in number, still occur with regularity. Depending on how these assessments are done, there is a concern that inter-clinician variability can impact the results, even in extreme ways (Cowan et al., 1994). These findings demonstrate the need for either (a) clear instruction and proof of inter-observer repeatability, like those shown in (Garrow et al., 2001), or (b) objective standards and quantitative methods of evaluating foot morphology.

### 4.2 How to measure

Two key aspects of foot measurement procedures are their objectivity and automation. The former aspect is important for the accuracy of the measurements obtained, and the latter is important as it impacts both the amount of effort and the costs involved in data collection.

Of the measurement styles reviewed, manual assessment is the most labor intensive as often, only one foot measurement is collected at a time. As a result, studies that employ manual measurement techniques often limit the number of measurements taken, leading to the potential of missing some relevant foot shape features. A manual procedure can also lead to examiner fatigue and loss of concentration, though the impact of this fatigue remains unclear. For example, Fryer et al. (2006) showed that examiner fatigue was not responsible for the low reliability when foot markers are assessed.

An additional concern with manual measurement procedures is the skill of the assessor. Studies have shown that using a measuring device for assessing joint range of motion (e.g., goniometer) can easily be affected by, among other things, the skill of the operator (Garrow et al., 2001). In addition, inter-observer variation is an issue with a variety of manually-collected measurements (Knippels et al., 2014). In summary, while manual foot shape assessment can be error prone and can have low precision, they are still widely-used due to their simplicity and low equipment cost.

For semi-automated measurement procedures, both physical and virtual markers are placed by experienced and well-trained professionals, which may introduce inter-assessor variability. Compared to physical markers, virtual markers usually reduce the measurement time for each subject (Krauss et al., 2011). However, physical markers allow palpation of the underlying bones on specific anatomical locations, something that virtual marker placement is unable to take into consideration. Consequently, physical markers are more accurate in annotating important features of the foot. That being said, it is recommended that physical markers should be placed onto the subject’s feet in a half-weight-bearing condition to limit skin movements between marker placement and scanning (Hill et al., 2017).

Alternatively, full automation of measurement procedures enables measurement of several foot dimensions at once, or even the capturing the whole foot shape in a fully numerical representation. This can be done with an automatic procedure because the procedure is often fast and highly accurate. Moreover, the digital representation of foot shape can be transmitted and recorded which removes the constraints of time, distance and/or use of second opinion. Nevertheless, automated procedures do have their downsides, one of which is the challenge involved in analyzing the large data sets that automated procedures can generate. For example, 3D optical scanners can produce a whole point cloud of thousands of pseudo-markers. In order to statistically analyse these pseudo-markers, it is necessary to establish geometrical correspondence between them so that all pseudo-markers refer to the same anatomical locations (Stanković et al., 2018). This correspondence task often requires advanced data analysis techniques.

It is also worth noting that, although the accuracy of data obtained by semi-automatic and automatic procedures is relatively high compared to manual procedures (Telfer and Woodburn, 2010), significant differences in foot length and foot width have been reported between manual foot measurements and measurements obtained using 3D foot scanners (Xiong et al., 2008). It has been shown that these differences are due to toe separation and toe flexion during scanning (Xiong et al., 2008).

Finally, one inevitable practical consideration for foot measurement collection is the cost involved. In terms of equipment costs, a manual procedure is obviously cheaper compared to semi-automatic and automatic procedures. While semi-automatic and automatic procedures are expensive in terms of equipment (e.g., requiring 3D scanners), they potentially have lower measurement costs due to less manual labour. At the same time, the large data set sizes created by automated procedures may require more laborious data processing and analysis. Finally, low cost equipment has been reported for the purposes of 3D scanning (Wu et al., 2018; Ma and Luximon, 2014; Rogati et al., 2019; Yuan et al., 2021) which may automated 3D scanning more accessible for automated foot shape measurement.

### 4.3 How to analyze

As can be observed in Table 2, there is no clear link between the choice of measurements and the type of analysis performed. Instead, the choice of analysis technique depends on the goal of the study (Table 3). For studies summarizing foot shape measurements, a technique like PCA is commonly used to describe foot shape variability in a given population. For studies aiming to divide a population into groups for designing different footwear for each group, classification and clustering techniques are utilized. Meanwhile, group studies can be used to see if different groups require different footwear or orthotic designs, while prediction methods are commonly used to estimate a more detailed foot shape from a smaller collection of measurements.

While the high-level analysis techniques are goal driven, there are a few considerations that are somewhat universal. Sample sizes and data distributions can influence a variety of these analysis techniques. Having a small sample size irrevocably leads one to not have much confidence in results based on very small sample sizes, irrespective of the analysis technique used. Meanwhile, a variety of analysis techniques assume normally distributed samples. These techniques include commonly-used approaches like t-tests, PCA, K-means, and linear regression. The effectiveness and reliability of these analyses can be questioned if the data used is not checked for normality.

It is also worth noting that more advanced analysis techniques exist that have yet to be exploited in the papers we reviewed. These include more advanced classification techniques (Kotsiantis et al., 2007) and artificial intelligence-based prediction models (Wu et al., 2018; Booth et al., 2019; Boppana and Anderson, 2021). The use of more advanced analysis techniques could provide better estimates of key features for footwear and orthotic design, and the application of these techniques is an interesting topic for future work.

### 4.4 What else can impact foot shape analysis

Table 4 shows that a variety of personal characteristics and lifestyle choices can influence one’s foot shape. Sex-based foot shape differences are the most supported by the studies we reviewed, and the takeaway message behind these studies are that a women’s foot cannot be considered a scaled down version of a man’s foot. While a women’s foot is generally narrower and smaller than a man’s foot, a women’s toe sizes, arch height, and metatarsal angles have been shown to be relatively higher than those of a man’s foot. As a result, it is recommended that the feet of males and females be analyzed separately, and that footwear and orthotic designs be different for the two sexes.

In addition to sex-based foot shape differences, there is evidence that older populations show differences in foot shape, specifically having wider feet and higher forefoot volumes. Therefore, an analysis of foot shape should keep in mind to include a suitable number of older individuals, and a design of orthotics for older populations should allow for wider feet. Similarly, individuals with a high BMI also show wider feet, which indicates that any foot shape analysis should be balanced or normalized for different BMIs. A similar point can be said for individuals with different ethnicities. These studies suggest that orthotic and footwear design can be improved by considering these personal characteristics.

From the point of view of orthotic design, it is worth keeping in mind that a variety of foot problems can change foot morphology. This can be a concern if an orthotic is meant for both temporary and corrective use. If an orthotic is providing the necessary support to enable healing, then the shape of the foot may change as a result of the healing process, resulting in a poorer fit between the orthotic and the foot. The design of orthotics that can adapt to changes in foot shape may be an interesting area of future work.

Finally, lifestyle choices can also influence foot shape, specifically the use of high heels and physical activity. When designing a high heel shoe, evidence suggests that more room should be provided in the forefoot. The same conclusion can also be supported for athletic shoes, along with a narrower midfoot and heel. If you are designing footwear for these specific applications, or for populations that engage in these lifestyles, then studying the foot shapes from an average population may not provide one with foot shape measurements that are representative of the population of interest.

## 5 Conclusion

Numerous techniques for foot shape quantification have been reported for the purpose of footwear and orthotic design. As researchers seek to improve footwear and orthotics, a deeper understanding of how foot shape relates to different aspects of human physiology and biomechanics can be valuable. In this review, we observed three main foot measurement types (visual, anthropometric, and geometric) and three main measurement techniques (manual, semi-automatic, automatic). We observed a clear shift towards automatic foot shape measurement over the last years, as nowadays 3D scanners are becoming more accessible and provide a more complete measurement of foot shape. Additionally, we observed a variety of analysis techniques, and a variety of foot shape changes influenced by an individual’s characteristics (sex, age, ethnicity, BMI) or behavior (amount of physical activity). We therefore conclude that footwear and orthotic designs can be improved by incorporating these physical and behaviour characteristics into the foot shape analysis.

## Data Availability

The original contributions presented in the study are included in the article/Supplementary Material, further inquiries can be directed to the corresponding author.
