# Supplementary material for: Foot shape assessment techniques for orthotic and footwear applications: a methodological literature review
Source: Front Bioeng Biotechnol. 2024 Sep 5;12:1416499. doi: 10.3389/fbioe.2024.1416499 (PMC11410610; doi:10.3389/fbioe.2024.1416499)
Supplement: Supplementary file 1 [file DataSheet1.PDF]

frontiers

Supplementary Material

1 SUPPLEMENTARY DATA

Table S1: **Different types of foot measurements.** V-visual, G-geometrical, A-anthropometric measurements used for different orthotic and footwear applications. Occurrence in the reported studies: $> 10\%$*, $> 20\%$**, $> 30\%$***, $> 40\%$****. Foot variability (i.e., study of the main shape variations of the foot), subject features (i.e., study of the relationship between different foot measurements), subject behaviour or health condition (i.e., study of influence of behaviour or health on the shape of the foot), custom products (i.e., study to improve footwear products), definition of product sizes (i.e., study to improve the footwear sizes), new measurement system (i.e., study to improve the measuring system of feet.) Note that due to the wide range of studies and the variety of foot measurements, the occurrence of any single specific foot measurement never exceeded 50%.

	**Foot variability**	**Subject features**	**Subject behaviour or health condition**	**Custom products**	**Definition of product sizes**	**New measure. system**	**Other**
Footprint (G) *	(1; 2)	(3; 4; 5), (6; 2)		(7; 8)	(9)	(10)	(11)
Footprint angle (A)					(12)		
Footprint index (A)					(12)		
3D foot shape (G) **	(13; 14; 15), (16; 17; 18)	(19; 20)	(21; 7; 22)	(23; 24; 25)	(20; 26; 27), (28)	(29; 30)	
Foot length (A) ****	(31; 32; 1), (16; 33; 17)	(34; 35; 36), (37; 38; 39), (40; 41; 30), (42)	(43; 30; 44), (45; 21)	(23; 43; 46), (7)	(12; 47)	(29; 48; 28)	(11; 30)
Foot width (A) **	(1; 16; 17), (31)	(34; 37; 32), (36; 49; 50), (41)	(44; 43)	(7; 43; 46)	(12)	(29; 28)	(30)
Foot circumf. (A)	(31)						
Ball angle (A) *		(51; 52; 37), (38; 53)	(21)		(12)		(11)
Ball circumf. (A) ****	(16; 54; 33)	(34), (35; 36), (55; 39; 40), (56; 53; 32), (49; 57; 42)	(43; 58; 59), (45; 21)	(46; 43; 7)	(12)	(29; 48)	(11; 30)

Ball height (A) *		(34; 55; 39), (56; 49; 57), (42)	(58; 59; 21)				
Ball length (A) *		(52; 39; 53), (49)	(21)	(46)			(11)
Ball-to-heel length (A)		(49)					(11)
Ball width (A) **	(54; 33)	(34; 35; 42), (52; 55; 40), (56; 53; 57)	(58; 59; 21)		(12)	(48)	(11)
Height of top of ball circumf. (A)		(49)		(43)			
Instep circumf. (A) **	(54)	(35; 36; 55), (39; 56; 49), (57; 42)	(58; 59; 45; 21)	(43; 46; 7)	(12)	(29; 48)	
Instep height (A) **	(31; 54; 33)	(36; 42; 34), (35; 52; 37), (55; 38; 39), (53)	(45; 59; 21)	(43; 46)	(47)	(48)	
Instep length (A) *		(34; 36; 52), (37; 38; 49)	(43)	(43)	(47)		(30)
Instep size (A)							(11)
Instep width (A)		(53; 42; 31)					
Plantar circumf. (A)				(23)			
Medial metatarsal length (A) *		(34; 51; 37), (55; 38; 36)		(43)	(12)	(29)	(30)
Lateral metatarsal length (A) *		(34; 51; 37), (55; 38; 36), (52; 49)	(43)	(43)	(12; 47)	(29)	(30)
Heel circumf. (A) *		(34; 36; 40), (49)	(59)	(7)			(11)
Heel length (A)					(12)		
Heel width (A) ***	(16)	(34; 35; 51), (52; 37; 55), (38; 56; 53), (42; 36; 49)	(59; 45; 21), (43)	(43; 46)	(12)	(29)	(11; 48)
Arch angle (A)	(1)	(51)	(60)				
Arch circumf. (A)		(40)			(12)		

Arch height (A) *	(1)	(4; 51; 49) (61; 62)	(58; 59)	(46)	(12)	(63; 28)	
Arch index (A) *	(1)	(4; 51; 56), (5; 57)	(58; 59; 64)			(63)	
Arch length (A)	(16; 17)	(34)			(12)	(29; 48)	
Arch width (A)	(16)	(51)			(12)	(63; 28)	(11)
Dorsal arch circumf. (A)					(12)		(11)
Dorsal arch height (A)		(56; 49)	(60)		(12; 47)		(11)
Foot slope (A)							(11)
Navicular height (A) *	(33)	(35; 55)	(60; 64; 65), (45)	(43)	(47)		
Medial malleolus height (A) *	(66)	(37; 55; 53), (49)	(59)			(29)	
Lateral malleolus heigth (A)		(37; 55; 53), (49)				(29)	
Midfoot circumf. (A)					(12)		
Midfoot height (A)						(29)	
Midfoot width (A)	(16)					(29)	
Ankle circumf. (A)		(57)	(59)	(7)	(12)		
Sphyrion height (A)		(36; 37)					
Toe #1 angle (A) *		(35; 36; 55), (56; 57)	(44; 58; 59), (21)	(43)			
Toe #5 angle (A) *		(35; 36; 55), (56; 57)	(58; 21)	(43)		(48)	
Toe #1 height (A) *		(35; 37; 55), (38; 39; 56), (53; 49; 57)	(58; 59)	(46)			
Toe #5 height (A)		(35; 37; 55)					
Toe #1-#5 circumf. (A)		(56; 57)	(58; 59)				
Toe #1-#5 width (A)		(56; 57)	(58; 59)				
Toes height (A)	(54)	(49)					(11)

Truncated foot length (A)	(66)	(35)			(47)		
Foot posture index (V) *		(56; 57)	(58; 59; 67), (64; 65)			(28)	
Hallux valgus index (A)							(68)
Hallux valgus scale (V)						(69)	
Minimal distance between hallux and the interphalangeal joint of the second toes (A)			(44)				

Table S2. Studies that are using different automation levels of measurement procedure related to the number of measured foot shape characteristics (* virtual markers, ’ physical markers).

	Manual	Semi-automatic	Automatic
**Foot lengths**	(11), (23), (40), (53), (49), (50), (57), (41), (48), (28)	(43)’, (30)*, (35)’, (36)’, (46)’, (51)*, (52)*, (37)’, (55)’, (38)’, (31)’, (39)’, (44)’, (32)’, (1)’, (47)’, (54)’, (45)’, (66)*, (18)’, (21)*, (8)*	(34), (12), (29), (33), (21), (28)
**Foot widths**	(11), (63), (40), (56), (58), (59), (53), (49), (50), (57), (41), (48), (28)	(43)’, (30)*, (35)’, (36)’, (46)’, (51)*, (52)*, (37)’, (55)’, (38)’, (31)’, (44)’, (32)’, (1)’, (54)’, (45)’, (18)’, (21)*, (8)*	(34), (12), (29), (33), (21), (28)
**Foot heights**	(11), (4), (63), (56), (58), (59), (53), (61), (62), (49), (57), (60), (64), (65), (48), (28)	(43)’, (35)’, (36)’, (51)*, (52)*, (37)’, (55)’, (38)’, (31)’, (39)’, (1)’, (47)’, (54)’, (45)’, (66)*, (18)’, (21)*, (8)*	(34), (12), (29), (33), (21), (28)
**Foot circumferences**	(11), (23), (40), (56), (58), (59), (53), (49) (57), (48)	(43)’, (30)*, (35)’, (36)’, (46)’, (55)’, (31)’, (39)’, (32)’, (54)’, (45)’, (18)’, (21)*, (8)*	(34), (12), (29), (33), (21)
**Foot angles**	(11), (56), (58), (59), (53), (57), (60)	(43)’, (35)’, (36)’, (51)*, (52)*, (37)’, (55)’, (38)’, (44)’, (1)’, (18)’, (21)*, (8)*	(12), (21)
**Foot indexes**	(4), (63), (56), (58), (59), (67), (57), (60), (64), (65)	(51)*, (1)’	(12), (5), (70)
**Plantar surface**			(71), (3), (11), (4), (63), (5), (6), (2), (9), (10), (28)
**3D foot shape**			(13), (15), (23), (19), (14), (24), (20), (16), (29), (72), (25), (27), (17), (21), (22), (28)

Table S3. Foot shape analysis methods for orthotic and footwear applications.

	Foot shape variation	Group studies	Prediction	Classification and clustering
**Describe foot variation**	(71), (13), (15), (31), (14), (16), (2)	(1)	(32), (17)	(54), (33), (66), (18)
**Linking to subject characteristics**	(3), (6), (70)	(34), (35), (36), (51), (52), (37), (55), (38), (19), (56), (53), (32), (5), (49), (50), (20), (2), (41), (42), (18)	(4), (40), (19), (49)	(37), (38), (39), (61), (62)
**Linking to subject behaviour and health condition**		(43), (55), (44), (58), (59), (67), (57), (60), (64), (65), (45), (22)		
**Custom products**	(8)	(23), (25)	(24)	(43), (46)
**Definition of product sizes**	(7)	(20)	(47)	(12), (26), (9), (27)
**New measurement system**	(21)	(63), (29), (72), (10)		(69), (48)

Table S4. List of studies that evaluate foot shape changes due to factors influence.

Technique	Factor	Study
Regression	Age	(13), (40), (70)
	Gender	(3), (13), (70)
	BMI	(3), (13), (51), (40), (70)
	High-heeled shoes	(71)
	Frequency of sport activity	(13)
Principal component regression	Age	(14)
	Gender	(14)
	BMI	(14)

Table S5. List of studies that employ prediction techniques to estimate predictive significance of related subject characteristics.

Technique	Predictor	Study
Regression	Age	(40)
	BMI	(19)
	Arch height	(4)
	Hallux valgus index	(68)
	Foot length	(47)
Principal component regression	Age	(14)
	Gender	(14)
	BMI	(14)
Machine learning	10 foot dimensions	(24)
Allometry	Foot length	(32)

REFERENCES

[1]B. Y. S. Tsung, M. Zhang, Y. B. Fan, D. A. Boone, et al., Quantitative comparison of plantar foot shapes under different weight-bearing conditions, J Rehabil Res Dev (2003).

[2]C. Sforza, G. Michielon, N. Fragnito, V. Ferrario, Foot asymmetry in healthy adults: elliptic fourier analysis of standardized footprints, Journal of Orthopaedic Research 16 (6) (1998) 758–765 (1998).

[3]J. Domjanic, H. Seidler, P. Mitteroecker, A combined morphometric analysis of foot form and its association with sex, stature, and body mass, American Journal of Physical Anthropology 157 (4) (2015) 582–591 (2015).

[4]M. R. Hawes, W. Nachbauer, D. Sovak, B. M. Nigg, Footprint parameters as a measure of arch height, Foot Ankle 13 (1) (1992) 22–26 (1992).

[5]N. Stolwijk, J. Duysens, J. Louwerens, Y. Ven, N. Keijsers, Flat feet, happy feet? comparison of the dynamic plantar pressure distribution and static medial foot geometry between malawian and dutch adults, PLoS One 8 (2) (2013) 57209 (2013).

[6]K. Jelen, Z. Tetkova, L. Halounova, K. Pavelka, T. Koudelka, P. Ruzicka, Shape characteristics of the foot arch: dynamics in the pregnancy period, Neuroendocrinology Letters 26 (6) (2005) 752–756 (2005).

[7]L. Chertenko, B. Booth, Modelling shape and parameterising style: an approach to the design of high-fashion shoe lasts, Footwear Science 14 (2022) 1–20 (07 2022).

[8]S. Bogdan, A. Mihai, M. Costea, E. Rezus, Comparative anthropometric study regarding the foot of elderly female population, Procedia Engineering 181 (2017) 182–186 (12 2017).

Table S6. List of studies that employ classification techniques to distinct foot shape groups.

Technique	Classification groups	Study
Achetypoid analysis	The first archetypoid is a very short and narrow foot; the second archetypoid is very wide foot; and the third archetypoid is a very long foot	(54)
Archetype analysis + knn	Normal shape or outlier	(33)
Discriminant analysis	Recreational sprinters and non-habitual exercisers	(43)
	Gender	(49)
Free form deformation method	Four groups: (a) short toes and large leg depth; (b) long toes and small leg depth; (c) low dorsal and plantar arch; (d) high dorsal arch	(26)
Calculation of central tendencies based of 4 plantar shape parameters: foot width, heel width, three-quarters of the length of the foot; the arch height	S, M, L types based on their central tendencies	(9)
Mean and standard deviation of truncated normalized navicular height are used as the limits for group ranges	Arch height (normal, pes cavus, pes planus)	(66)
Visual appraisal	Arch height (normal, pes cavus, pes planus)	(61), (62)
	Hallux valgus (no, mild, moderate, severe)	(69)

[9]S. Sun, Y. Chou, C. Sue, Classification and mass production technique for three-quarter shoe insoles using non-weight-bearing plantar shapes, Applied Ergonomics 40 (4) (2009) 630–635 (2009).

[10]G. Rogati, A. Leardini, M. Ortolani, P. Caravaggi, Validation of a novel Kinect-based device for 3d scanning of the foot plantar surface in weight-bearing, Journal of Foot and Ankle Research 12 (1) (2019) 1–8 (2019).

[11]F. L. Bookstein, J. Domjanić, Analysis of the human female foot in two different measurement systems: From geometric morphometrics to functional morphology, Collegium antropologicum 38 (3) (2014) 855–863 (2014).

[12]S.-Y. Baek, K. Lee, Statistical foot-shape analysis for mass-customisation of footwear, International Journal of Computer Aided Engineering and Technology 8 (1-2) (2016) 80–98 (2016).

[13]K. Stanković, B. G. Booth, F. Danckaers, F. Burg, P. Vermaelen, S. Duerinck, J. Sijbers, T. Huysmans, Three-dimensional quantitative analysis of healthy foot shape: a proof of concept study, Journal of Foot and Ankle Research 11 (1) (2018) 1–13 (2018).

[14]K. Stanković, T. Huysmans, F. Danckaers, J. Sijbers, B. Booth, Subject-specific identification of three dimensional foot shape deviations using statistical shape analysis, Expert Systems with Applications 151 (2020) 113372 (2020).

[15]B. P. Conrad, M. Amos, I. Sintini, B. R. Polasek, P. Laz, Statistical shape modelling describes anatomic variation in the foot, Footwear Science 11 (sup1) (2019) S203–S205 (2019).

[16]G. Park, R. Kent, Foot shape analysis of professional american football players, Footwear Science 12 (3) (2020) 153–159 (2020).

Table S7. List of studies that apply comparison techniques for evaluation of measurement system based on extracted foot measurements.

Technique	Measurement	Application	Study
MSE	Several foot dimensions	Comparison of proposed low cost 3D scanning procedure to the measurement procedure which uses anthropometric data	(23)
	Several foot dimensions	Comparison of scanned and predicted foot shape for the purposes of the shape reconstruction	(29)
	Foot outline, foot profile, several sections	Comparison of scanned and predicted foot shape for the purposes of using low cost scanning	(72)
RMSE	Plantar foot shape	Comparison of 3D foot high resolution scan and low cost 3D foot scan for the purposes of using low cost scanning	(10)
	3D foot shape	Comparison of scanned and predicted foot shape for the purposes of shape reconstruction	(17)
ICC	Several foot dimensions	Comparison of measurements derived from semi-automatic and automatic landmark detection	(21)
Data distribution measurements	Several foot dimensions	Comparison of 3D Fourier descriptor foot model to the homologous model	(30)
Spearman's rank order correlation	Foot parameter (arch index) and arch dimensions	Comparison of estimated results to the ground truth	(63)
Linear regression	Single foot dimension (arch width)	Comparison between two different measurement systems based on the arch width as a common variable for both data sets	(36)
Mean Euclidean distance	3D foot shape	Comparison of scanned and predicted foot shape for the purposes of the shape reconstruction	(29)
Hausdorff distance	3D foot shape	Comparison of scanned and predicted foot shape for the purposes of the shape reconstruction	(17)
Mean shortest distance between shapes	3D foot shape	Comparison of actual scan and predicted foot parameterized using foot length, foot width, foot height, and a measure of foot curvature so that foot shape for the generation of personalized last	(25)

[17]A. Boppana, A. P. Anderson, Dynamic foot morphology explained through 4d scanning and shape modeling, Journal of Biomechanics 122 (2021).

[18]B. Cao, J. Wang, W. Shi, X. Lu, K. Zhou, 3d foot anthropometric measurements under two weight-bearing conditions for ergonomic design of foot-related products, International Journal of Morphology

Table S8. Papers showing which factors influence specific foot regions.

	Age	BMI	Ethnicity	Foot problems	Sex	Others
**Toes**	(13), (56)		(50)	Hallux valgus- (14); Hallux valgus, Toe deformity, Swollen foot - (55)	(3), (13), (35), (56), (49), (18)	High-heeled shoes - (71); Frequency of sport activity- (13), (43); Different bearing weight- (41); Shod and unshod runners- (44)
**Forefoot**	(56)	(13), (11), (34), (51), (19)	(36), (46), (50)	Hallux valgus - (14); Hallux valgus, Toe deformity, Swollen foot - (55)	(46), (52), (37), (55), (38), (53), (32), (50), (18)	High-heeled shoes - (71); Geographic region- (20); Frequency of sport activity- (43); Different bearing weight- (41); Shod and unshod runners- (44)
**Midfoot**	(40), (70)	(71), (3), (11), (34), (51), (40), (19), (70)	(5), (50)	Hallux valgus, Toe deformity- (55); Patellofemoral pain syndrome- (60); Osteoarthritis- (64)	(3), (13), (35), (46), (52), (37), (55), (38), (56), (49), (20), (18), (70)	Frequency of sport activity- (13); Geographic region- (20)
**Heel**	(13)	(13), (11), (34)	(50)	Swollen foot- (55) Patellofemoral pain syndrome- (60); Arthritis- (57); Osteoarthritis- (64); Diabetic foot- (67)	(13), (46), (56), (20), (18)	
**Ankle**	(13)	(13), (11)	(50)	Toe deformity, Swollen foot- (55) Patellofemoral pain syndrome- (60); Arthritis- (57); Osteoarthritis- (64); Diabetic foot- (67)	(13), (56), (49), (18)	Frequency of sport activity- (13)

41 (2023) 1209–1218 (08 2023).

[19]W.-K. Chiou, H.-T. Chiu, A.-S. Chao, M.-H. Wang, Y.-L. Chen, The influence of body mass on foot dimensions during pregnancy, Applied Ergonomics 46 (2015) 212–217 (2015).

[20]A. Jurca, J. Žabkar, S. Džeroski, Analysis of 1.2 million foot scans from North America, Europe and Asia, Scientific Reports 9 (1) (2019) 1–10 (2019).

[21]L.-Y. Zhang, K.-L. Yick, M.-j. Yue, J. Yip, Z. Ng, An exploratory study of dynamic foot shape measurements with 4d scanning system, Scientific Reports 13 (05 2023).

[22]R. W. Schuster, A. Cresswell, L. Kelly, Reliability and quality of statistical shape and deformation models constructed from optical foot scans, Journal of Biomechanics 115 (2021) 110137 (2021).

[23]M. Wang, X. Wang, Z. Fan, S. Zhang, C. Peng, Z. Liu, A 3D foot shape feature parameter measurement algorithm based on kinect2, EURASIP Journal on Image and Video Processing 2018 (1) (2018) 1–12 (2018).

[24]B. Booth, J. Sijbers, T. Huysmans, A machine learning approach to the design of customized shoe lasts, Footwear Science 11 (sup1) (2019) 17– 19 (2019). doi:10.1080/19424280.2019.1606055.

[25]A. Luximon, R. Goonetilleke, Foot shape modeling, Human Factors 46 (2) (2004) 304–315 (2004).

[26]M. Mochimaru, M. Kouchi, M. Dohi, Analysis of 3-D human foot forms using the free form deformation method and its application in grading shoe lasts, Ergonomics 43 (9) (2000) 1301–1313 (2000).

[27]S. Huang, Z. Wang, Y. Jiang, Guess your size: A hybrid model for footwear size recommendation, Advanced Engineering Informatics 36 (2018) 64–75 (2018).

[28]G. Rogati, A. Leardini, M. Ortolani, P. Caravaggi, Semi-automatic measurements of foot morphological parameters from 3d plantar foot scans, Journal of Foot and Ankle Research 14 (2021).

[29]G. Wu, D. Li, P. Hu, Y. Zhong, N. Pan, Foot shape prediction using elliptical Fourier analysis, Textile Research Journal 88 (9) (2018) 1026–1037 (2018).

[30]O. M. Rijal, M. F. M. Hamzah, S. Sankaraiah, N. M. Noor, A three dimensional foot fourier descriptors model, ARPN Journal of Engineering and Applied Sciences 13 (13) (2018) 4042–4056 (2018).

[31]M. Kouchi, E. Tsutsumi, Relation between the medial axis of the foot outline and 3-D foot shape, Ergonomics 39 (6) (1996) 853–861 (1996).

[32]S. Xiong, R. S. Goonetilleke, C. P. Witana, E. Y. Lee Au, Modelling foot height and foot shape-related dimensions, Ergonomics 51 (8) (2008) 1272–1289 (2008).

[33]I. Cabero, I. Epifanio, A. Piérola, A. Ballester, Archetype analysis: A new subspace outlier detection approach, Knowledge-Based Systems 217 (2021) 106830 (2021).

[34]C. Price, C. Nester, Foot dimensions and morphology in healthy weight, overweight and obese males, Clinical Biomechanics 37 (2016) 125–130 (2016).

[35]M. Saghazadeh, N. Kitano, T. Okura, Gender differences of foot characteristics in older Japanese adults using a 3D foot scanner, Journal of Foot and Ankle Research 8 (1) (2015) 1–7 (2015).

[36]Y.-C. Lee, M. Kouchi, M. Mochimaru, M.-J. Wang, Comparing 3D foot shape models between Taiwanese and Japanese females, Journal of Human Ergology 44 (1) (2015) 11–20 (2015).

[37]Y. Hong, L. Wang, D. Q. Xu, J. X. Li, Gender differences in foot shape: a study of Chinese young adults, Sports Biomechanics 10 (02) (2011) 85–97 (2011).

[38]I. Krauss, S. Grau, M. Mauch, C. Maiwald, T. Horstmann, Sex-related differences in foot shape, Ergonomics 51 (11) (2008) 1693–1709 (2008).

[39]N. Kim, W. Do, Developing elderly men's footwear sizing system based on their foot shapes, Fashion and Textiles 6 (1) (2019) 1–18 (2019).

[40]J. A. Echeita, J. M. Hijmans, S. Smits, L. H. Van der Woude, K. Postema, Age-related differences in women's foot shape, Maturitas 94 (2016) 64–69 (2016).

[41]G. Oladipo, I. Bob-Manuel, G. Ezenatein, Quantitative comparison of foot anthropometry under different weight bearing conditions amongst Nigerians, Internet J Bio Anthrop 3 (1) (2009).

[42]A. Ballester, A. Piérola, C. Solves-Camallonga, E. Parrilla, J. Uriel, I. Zaimi, S. Alemany, Study on controllable and uncontrollable factors affecting foot shape, Footwear Science 11 (sup1) (2019) 123–125 (2019).

[43]L.-H. Chen, C.-C. Chang, M.-J. Wang, L. Tsao, Comparison of foot shape between recreational sprinters and non-habitual exercisers using 3D scanning data, International Journal of Industrial Ergonomics 68 (2018) 337–343 (2018).

[44]Y. Shu, Q. Mei, J. Fernandez, Z. Li, N. Feng, Y. Gu, Foot morphological difference between habitually shod and unshod runners, PloS One 10 (7) (2015) e0131385 (2015).

[45]C. Maiwald, T. Mayer, T. Milani, Alterations of plantar pressure patterns and foot shape after long distance military marching, Footwear Science 10 (3) (2018) 203–213 (2018).

[46]Y.-C. Lee, M.-J. Wang, Taiwanese adult foot shape classification using 3D scanning data, Ergonomics 58 (3) (2015) 513–523 (2015).

[47]M. Hill, R. Naemi, H. Branthwaite, N. Chockalingam, The relationship between arch height and foot length: Implications for size grading, Applied Ergonomics 59 (2017) 243–250 (2017).

[48]M. K. Limon, M. Uddin, M. Hossin, M. Rahman, Development of new shoe sizing system for women based on regression analysis of foot shapes, International Journal of Industrial Ergonomics 94 (2023) 103408 (2023).

[49]R. Wunderlich, P. Cavanagh, Gender differences in adult foot shape: implications for shoe design, Medicine and Science in Sports and Exercise 33 (4) (2001) 605–611 (2001).

[50]I. Bob-Manuel, B. Didia, Sexual dimorphism in foot dimensions among adult Nigerians, Internet J Biol Anthropol 3 (1) (2009) 1–6 (2009).

[51]B. Fritz, T. Schmeltzpfenning, C. Plank, T. Hein, S. Grau, Anthropometric influences on dynamic foot shape: measurements of plantar three-dimensional foot deformation, Footwear Science 5 (2) (2013) 121–129 (2013).

[52]I. Krauss, C. Langbein, T. Horstmann, S. Grau, Sex-related differences in foot shape of adult caucasians–a follow-up study focusing on long and short feet, Ergonomics 54 (3) (2011) 294–300 (2011).

[53]G. Luo, V. L. Houston, M. Mussman, M. Garbarini, A. C. Beattie, C. Thongpop, Comparison of male and female foot shape, Journal of the American Podiatric Medical Association 99 (5) (2009) 383–390 (2009).

[54]A. Alcacer, I. Epifanio, M. Ibanez, A. Simo, A. Ballester, A data-driven classification of 3D foot types by archetypal shapes based on landmarks, Plos One 15 (1) (2020) 0228016 (2020).

[55]K. J. Mickle, B. J. Munro, S. R. Lord, H. B. Menz, J. R. Steele, Foot shape of older people: implications for shoe design, Footwear Science 2 (3) (2010) 131–139 (2010).

[56]A. P. De Castro, J. R. Rebelatto, T. R. Aurichio, The effect of gender on foot anthropometrics in older people, Journal of Sport Rehabilitation 20 (3) (2011) 277–286 (2011).

[57]A. Castro, J. Rebelatto, T. Aurichio, P. Greve, The influence of arthritis on the anthropometric parameters of the feet of older women, Archives of Gerontology and Geriatrics 50 (2) (2010) 136–139 (2010).

[58]A. P. de Castro, J. R. Rebelatto, T. R. Aurichio, The relationship between foot pain, anthropometric variables and footwear among older people, Applied Ergonomics 41 (1) (2010) 93–97 (2010).

[59]A. P. de Castro, J. R. Rebelatto, T. R. Aurichio, The relationship between wearing incorrectly sized shoes and foot dimensions, foot pain, and diabetes, Journal of Sport Rehabilitation 19 (2) (2010) 214–225 (2010).

[60]C. Barton, D. Bonanno, P. Levinger, H. Menz, Foot and ankle characteristics in patellofemoral pain syndrome: a case control and reliability study, Journal of Orthopaedic & Sports Physical Therapy 40 (5) (2010) 286–296 (2010).

[61]D. Swedler, J. Knapik, T. Grier, B. Jones, Validity of plantar surface visual assessment as an estimate of foot arch height, Medicine and Science in Sports and Exercise 42 (2) (2010) 375–380 (2010).

[62]H. Menz, M. Fotoohabadi, E. Wee, M. Spink, Visual categorisation of the arch index: a simplified measure of foot posture in older people, Journal of Foot and Ankle Research 5 (1) (2012) 1–7 (2012).

[63]S. Chun, S. Kong, K.-R. Mun, J. Kim, A foot-arch parameter measurement system using a RGB-D camera, Sensors 17 (8) (2017) 1796 (2017).

[64]P. Levinger, H. Menz, M. Fotoohabadi, J. Feller, J. Bartlett, N. Bergman, Foot posture in people with medial compartment knee osteoarthritis, Journal of Foot and Ankle Research 3 (1) (2010) 1–8 (2010).

[65]E. Cowley, J. Marsden, The effects of prolonged running on foot posture: a repeated measures study of half marathon runners using the foot posture index and navicular height, Journal of Foot and Ankle Research 6 (1) (2013) 1–7 (2013).

[66]C. Hu, A. Baca, M. Groeber, P. Dabnichki, Geometrical model for characterization of foot deformity using 3D imaging, IFAC-PapersOnLine 51 (2) (2018) 373–378 (2018).

[67]J. Young, Foot shape and asymmetry in the Charcot foot: Assessment using the foot posture index, Journal of the American Podiatric Medical Association 110 (1) (2020).

[68]C. Thomson, An investigation into the reliability of the valgus index and its validity as a clinical measurement, The Foot 4 (4) (1994) 191–197 (1994). doi:10.1016/0958-2592(94)90051-5.

[69]A. Garrow, A. Papageorgiou, A. Silman, E. Thomas, M. Jayson, G. Macfarlane, The grading of hallux valgus. the manchester scale, Journal of the American Podiatric Medical Association 91 (2) (2001) 74–78 (2001).
[70]X. Zhao, Y. Gu, J. Yu, Y. Ma, Z. Zhou, The influence of gender, age, and body mass index on arch height and arch stiffness, The Journal of Foot and Ankle Surgery 59 (2) (2020) 298–302 (2020).
[71]J. Domjanic, M. Fieder, H. Seidler, P. Mitteroecker, Geometric morphometric footprint analysis of young women, Journal of Foot and Ankle Research 6 (1) (2013) 1–8 (2013).
[72]X. Ma, A. Luximon, 3D foot prediction method for low cost scanning, International Journal of Industrial Ergonomics 44 (6) (2014) 866–873 (2014).
